# Integrating data from spontaneous and induced *trans*-10 shift of ruminal biohydrogenation reveals discriminant bacterial community changes at the OTU level

**DOI:** 10.3389/fmicb.2022.1012341

**Published:** 2023-01-06

**Authors:** Francis Enjalbert, Asma Zened, Laurent Cauquil, Annabelle Meynadier

**Affiliations:** GenPhySE, Université de Toulouse, INRAE, ENVT, INPT, Toulouse, France

**Keywords:** ruminal bacteria, dairy cow, biohydrogenation, *trans*-10 fatty acids, milk fat depression

## Abstract

**Introduction:**

Microbial digestion is of key importance for ruminants, and disturbances can affect efficiency and quality of products for human consumers. Ruminal biohydrogenation of dietary unsaturated fatty acids leads to a wide variety of specific fatty acids. Some dietary conditions can affect the pathways of this transformation, leading to *trans*-10 fatty acids rather than the more usual *trans*-11 fatty acids, this change resulting in milk fat depression in dairy cows.

**Materials and methods:**

We combined data from an induced and spontaneous *trans*-10 shift of ruminal biohydrogenation, providing new insight on bacterial changes at different taxonomic levels. A *trans*-10 shift was induced using dietary addition of concentrate and/or unsaturated fat, and the spontaneous milk fat depression was observed in a commercial dairy herd.

**Results and discussion:**

Most changes of microbial community related to bacteria that are not known to be involved in the biohydrogenation process, suggesting that the *trans*-10 shift may represent the biochemical marker of a wide change of bacterial community. At OTU level, sparse discriminant analysis revealed strong associations between this change of biohydrogenation pathway and some taxa, especially three taxa belonging to *[Eubacterium] coprostanoligenes group*, *Muribaculaceae* and *Lachnospiraceae NK3A20 group*, that could both be microbial markers of this disturbance and candidates for studies relative to their ability to produce *trans*-10 fatty acids.

## Introduction

Ruminants are highly dependent on ruminal microbiota, which degrades carbohydrates to short chain fatty acids (FA), transforms the majority of dietary nitrogen to microbial proteins, biohydrogenates unsaturated FA and synthesizes vitamins. As a result of this predominant effect on dietary constituents, disturbances of ruminal function can affect health, feed efficiency and quality of products from farm animals. Milk fat depression (MFD) is characterized by a reduction of milk fat content and yield in dairy cows. Although shifts of ruminal short chain FA profile can result in moderate MFD (Lee et al., 2021), the most prevalent hypothesis to explain strong MFD is a shift of biohydrogenation (BH) of linoleic acid (LA; cis-9,cis-12-C18:2) from a *trans*-11 (t11) to a *trans*-10 (t10) pathway (Griinari et al., 1998), with a key BH intermediate, *trans*-10, *cis*-12 conjugated linoleic acid (t10c12 CLA). This isomer is a strong inhibitor of lipogenesis in the mammary gland (Baumgard et al., 2001), is exported into milk and promotes atherosclerosis in mice (Arbonés-Mainar et al., 2006). Experimental data including induction and recovery studies (Piperova et al., 2002; Weimer et al., 2010; Rico and Harvatine, 2013; Zened et al., 2013b; Pitta et al., 2020) show that the BH shift designated as t10-shift is linked with high dietary fermentable starch and unsaturated FA, and is associated with changes in the bacterial community. However, high starch or high fat diet can induce ruminal microbiota changes without t10-shift (Enjalbert et al., 2017; Plaizier et al., 2017a), which makes it difficult to ascertain what changes are specific to the t10-shift in MFD induction studies.

In culture studies, strains of *Megasphaera elsdenii* (Kim et al., 2002) and *Cutibacterium acnes* (formerly *Propionibacterium acnes*) (Wallace et al., 2007) have been shown to be able to synthesize t10c12 CLA from LA, but their implication in the t10-shift is somewhat controversial because other studies failed to reproduce this capacity for *M. elsdenii* (Maia et al., 2007), or studies concluded that it is unlikely that *C. acnes* is the only or predominant species involved in the t10-shift *in vivo* (Dewanckele et al., 2020). However, culture studies are conducted with available strains, and strain specific variations have been described in biohydrogenating bacteria (Hussain et al., 2016), so that results from such studies can fail to fit with bacterial community changes assessed *in vivo*.

Relationship between t10-shift and ruminal microbiota have only been studied in experimental conditions, reflecting the effects of heavy dietary changes after a few days or weeks adaptation (Weimer et al., 2010; Zened et al., 2013a,b; Pitta et al., 2018, 2020). In commercial dairy herds, MFD can also be observed in usual feeding conditions and during long periods, and differently affects cows receiving the same diet, reflecting host susceptibility (Conte et al., 2018). As a result, induced and spontaneous MFD could be associated with different microbiota changes. In a previous induction study we investigated changes of ruminal FA (Zened et al., 2013b) and microbiota at phylum to general levels (Zened et al., 2013a) after adaptation of cows. The present study combines data obtain during and after adaptation of cows in this experimental induction of t10-shift and data from a spontaneous MFD in a commercial dairy herd, in order to unravel common discriminant bacterial signatures associated with the t10-shift, at different taxonomic levels including OTU level.

## Materials and methods

### Animals and experiments

The experimental study used four dry ruminally fistulated Holstein cows that were assigned to a 4 × 4 Latin square design with four 14-days periods separated by two washout weeks, and four different diets based on corn silage and soybean meal. These diets combined two levels of concentrate (20 and 65% on a dry matter basis) and starch (20 and 34%,) obtained *via* addition of a wheat barley mixture, and two levels of fat (2.8 and 7.5%) obtained by sunflower oil addition. Diets, housing and design have been described in detail by Zened et al. (2013b). One liter of ruminal fluid was collected from each cow at 5 h post feeding at day 0 (last day of the washout weeks), day 2, day 8 and day 12 of each experimental period, resulting in 64 samples. Ruminal fluid was strained through a metal sieve (1.6-mm mesh). A 100 ml subsample was kept for FA determination and stored at −20°C, and 200 mg of filtrate were precisely weighed and stored in a 2-mL sterile Eppendorf tube at −80°C.

The field study was performed in a commercial dairy herd where part of cows exhibited a low milk fat content (33.3 ± 7.0 g/l; 16.5–51.8 range, measured by the dairy herd control laboratory). The 45 Holstein lactating cows with a 2.0 average parity received a total mixed ration providing nutrients for the average 32 kg milk production observed in the herd. The diet was based on corn silage and contained 37% concentrate, 24.5% starch and 3.7% fat (dry matter basis), offered as two equal meals at 07 h00 and 18 h00 and available *ad libitum*. Rumen and milk samples were taken on 22 cows, selected to be reflect the herd’s milk fat content (32.2 ± 7.2 g/l and 16.5–46.9 range for the selected cows). Ruminal fluid was taken by oral probe sampling between 13 h00 and 15 h00 on the same day for all cows, and was strained and stored as described for the experimental study. Milk was stored at −20°C until analysis.

### Analytical methods, 16S rRNA gene amplicon sequencing, and data transformation

Rumen and milk FA profiles were extracted, methylated, and analyzed by gas-chromatography as described by Zened et al. (2011). Briefly, GC analysis (Agilent 6,890 N, Network GC System, equipped with a model 7,683 autoinjector; Agilent Technologies Inc., Palo Alto, CA) was made on a fused silica capillary column (100 m × 0.25-mm i.d., 0.20-μm film thickness, CPSil88; Varian BV, Middelburg, Netherlands). Peaks were identified and quantified by comparison with commercial standards (Sigma Co., St Louis, MO), except C18:1 other than trans-9 C18:1, trans-11 C18:1, and cis-9 C18:1, which were identified by order of elution. The t10-shift of ruminal BH was assessed using the ruminal (t10 C18:1 + t10c12 CLA) / (t11 C18:1 + c9t11 CLA) ratio (t10t11R). Samples were classified as “Low,” “Medium,” or “High” t10t11R using 0.35 and 1.00 thresholds, that maximized area under curves calculated by sPLS-DA at OTU level, as described in the statistical analyses section.

DNA extraction and metabarcoding (using v3-v4 region of 16S RNA gene) of the 86 rumen samples were performed as described by Zened et al. (2013a), using 454 FLX pyrosequencing. Amplicon sequences were demultiplexed and then analyzed using the FROGS pipeline (Escudié et al., 2018). Firstly, they were filtered according to their size (400–500 nucleotides) and sequences presenting a primer mismatch or with one or more ambiguous base were removed as well as the chimeras. Then, kept sequences were clustered using Swarm with a defined difference of *d* = 1 between sequences in each aggregation step of clustering without exceeding a maximum of 3 different bases with the seed sequence. Finally, clusters with abundances <0.005% of the total number of sequences (Bokulich et al., 2013) were removed. Taxonomy assignment to OTU were done using the reference database Silva138 16S (Quast et al., 2013) with a pintail quality of 100. The mean number of sequences per sample was 2,834. OTUs with over 90% zeros in one of the two datasets or a maximal abundance across samples under 0.2% were filtered out. One sample with aberrant data (one OTU belonging to the *Bifidobacterium merycicum* species represented 44% of sequences) was discarded.

Because sequencing data have a compositional structure, they were transformed using the ZCompositions R package (Palarea-Albaladejo and Martín-Fernández, 2015) to imputed proportions by a Bayesian-multiplicative replacement of count-zeros (Martín-Fernández et al., 2015), further designated as GBM data. For statistical analyses, data were center log-ratio transformed (Quinn et al., 2019) and will be further designated as GBM-CLR data. GBM and GBM-CLR OTU abundances were agglomerated at the phylum, family, genus and species levels and items with an average relative abundance under 0.05% except 0.02% at species level in the three t10t11R classes were filtered out.

### Statistical analyses

Statistical analyses were performed using R-studio software, version 4.1.0. Alpha-diversity indexes were calculated on untransformed counts and were subjected to analysis of variance with fixed effects of study (field or induced), t10t11R class (Low, Medium or High) and their interaction, and the cow as a random effect. When the effect of t10t11R class was significant, a pairwise comparison was performed using the Tukey’s test.

Using GBM_CLR values, most discriminant genera and OTUs were selected using the MINT sPLS-DA procedure of the mixOmics R package (Rohart et al., 2017b). MINT is a multivariate method to integrate independent omics studies by simultaneously correcting for batch effects, classifying samples and identifying key discriminant variables (Rohart et al., 2017a). The number of components and the number of variables used to construct each component were chosen to maximize the area under curve calculated with the *perf* function. The outputs of mint.splsda analysis were used with the *network* function to plot relevance networks based on a similarity matrix, whose values can be seen as a robust approximation of the Pearson correlation (González et al., 2012), and to plot receiver operating characteristic curves using the auroc function.

Spearman correlations were calculated at different taxonomic ranks between the GBM-CLR abundances and the t10t11R, and were used for comparison with literature data. To evidence non-linear relationships, analyses of variances were performed with the same statistical model than alpha-diversity. An analysis of GBM data was used for determination of least-square means and their standard errors presented in tables, and GBM-CLR values were used for calculation of significances. *p*-values obtained from analysis of variance and correlation were adjusted for multi testing false discovery rate by the Benjamini−Hochberg procedure. *p* < 0.05 was considered significant.

## Results

**Figure 1:**
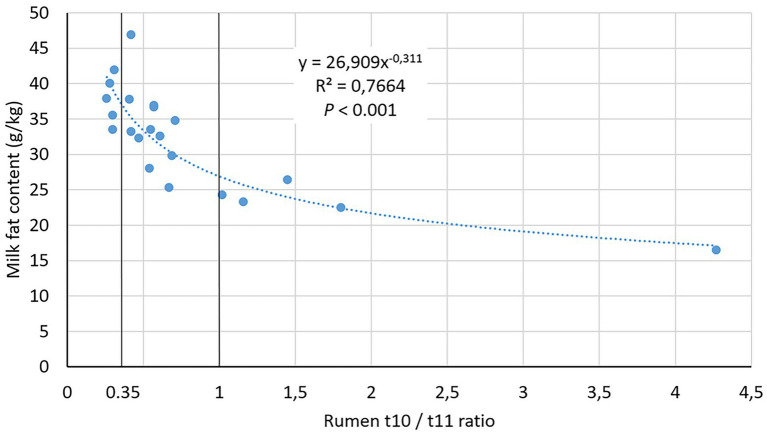
Relationship between rumen *trans*-10/*trans*-11 ratio and milk fat content in the field study.

Most samples from the induction study were in the Low t10t11R class (Supplementary Table 1), and rumen samples of the High class in the induction study exhibited a 7.97 t10t11R compared to 1.94 in the field study. In the field study, milk t10t11R were similar to ruminal ratios. Milk fat content was much lower in High than Low and Medium t10t11R classes (*p* < 0.001) and showed a strong nonlinear relationship with ruminal t10t11R (Figure 1). According to this relationship, our 0.35 and 1.0 thresholds for t10t11R classes corresponded with 37 and 27 g/kg milk fat content, respectively.

A total of 205,374 sequences regrouped into 1,185 OTUs were identified after filtering and clustering, 799 of them being shared by the two datasets. One OTU (unknown genus of *Lachnospiraceae*) was found in only three samples of the High t10t11R class whereas 162 OTUS were specific of the Low t10t11R class (Supplementary Figure 1). All sequences were assigned at phylum level (8 phyla), 98% were assigned to 42 families, 77% were assigned to 83 genera and 1.8% were assigned at species level (14 species). Abundances of main phyla, families, genera, and species across datasets and t10t11R classes are shown in Supplementary Figure 2. Among bacteria that are involved in BH or have been suggested to be involved in BH, only the *Butyrivibrio* genus (7 OTUs) was found in both datasets, and only one OTU could be affiliated at species level (*B. hungatei*). Alpha-diversity indexes were significantly lower in the Medium and High classes than in the Low class, and were lower in the induction study than in the field study (Table 1).

**Table 1:** Alpha-diversity of rumen microbiota across *trans*-10/*trans*-11 ratio classes and studies.

	*trans*-10 / *trans*-11 ratio class				Study			Interaction
	Low	Medium	High	*p*	Field	Induced	*p*	*p*
Observed	406^b^ ± 21	298^a^ ± 28	243^a^ ± 26	<0.001	366 ± 21	265 ± 20	<0.001	0.056
Shannon	5.05^b^ ± 0.10	4.44^a^ ± 0.13	4.04^a^ ± 0.12	<0.001	4.76 ± 0.10	4.25 ± 0.09	<0.001	0.015
Inv-Simpson	69.4^b^ ± 7.0	36.7^a^ ± 9.4	22.7^a^ ± 8.7	<0.001	48.7 ± 6.9	37.2 ± 6.7	<0.001	0.45

At phylum level, only *Desulfobacterota* and *Proteobacteria* were significantly correlated with the t10t11R (Supplementary Table 2), and significant differences between the t10t11R classes were only observed for *Desulfobacterota*, whose relative abundance was around 10 times higher in the High compared to the Low class. Significant correlation or significant differences between the t10t11R classes were observed in 18 families. The most important differences were observed for *Eubacteriaceae*, *Atopobiaceae*, *Veillonellaceae*, *Desulfovibrionaceae*, and *Lachnospiraceae*, whose relative abundances increased from the Low to the High class. *Bacteroidales RF16 group* relative abundance was higher in the Low than the Medium and High classes. Thirty genera and four species (Supplementary Table 3) and 201 OTUs (Supplementary Table 4) showed either significant correlation with t10t11R value or significant differences between t10t11R classes.

**Figure. 2:**
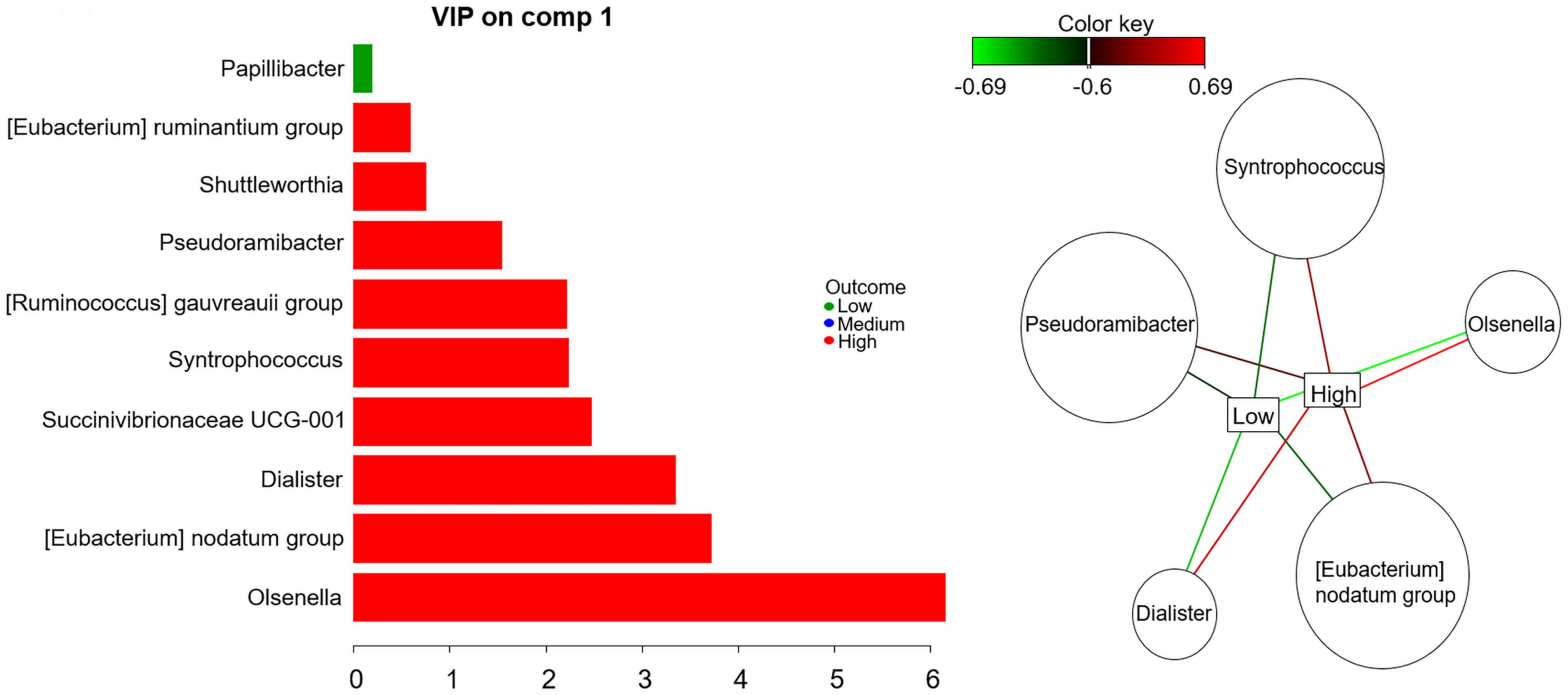
Mint sparse partial least square discriminant analysis on rumen bacterial genera: variable importance in projection (left) and relevance network with a 0.6 cut-off (right).

At genus level, the best performance of mint.sPLS-DA was obtained with one component and 10 genera. The component explained 13.5% of abundance variance. Figure 2 shows the variables importance in projection of the 10 genera. The relevance network evidenced that five of them were strongly (relevance network value >0.6) positively associated with high t10t11R and negatively associated with low t10t11R.

**Figure. 3:**
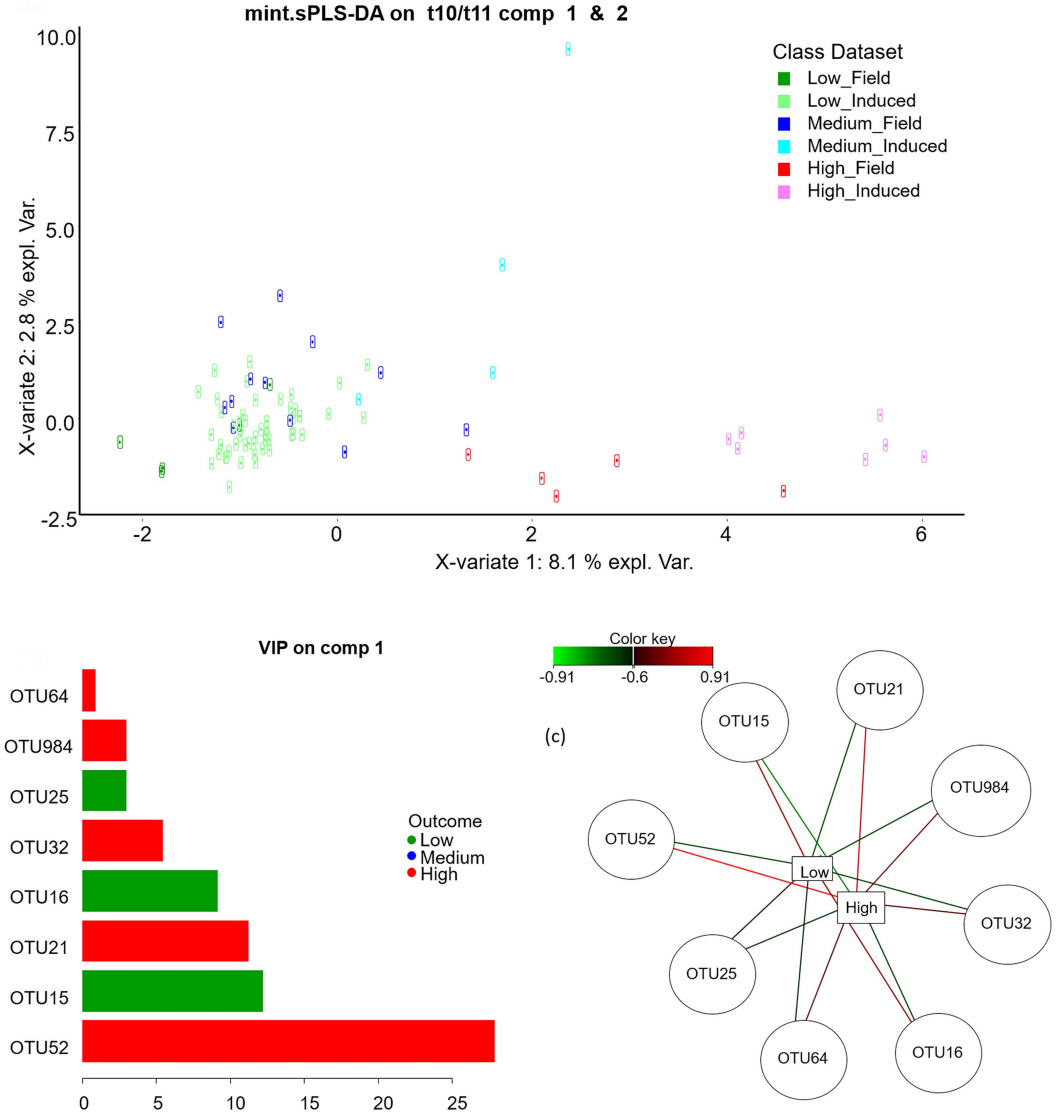
Mint sparse partial least square discriminant analysis on rumen bacterial OTUs: sample plot on the sPLS-DA components 1 and 2 (up), variable importance in projection on component 1 (bottom left) and relevance network with a 0.6 cut-off (bottom right).

At OTU level, the best mint.sPLS-DA performance was obtained with three components and eight, seven and one OTUs on components 1, 2, and 3, respectively. Component 1 discriminated between Low and High classes (Figure 3) whereas components 2 and 3 rather discriminated the Medium class. Five OTUs had a positive link with the High class, especially OTU52 as outlined by its 27.8 variable importance in projection (VIP) and its high opposed relations with High (value = 0.91) and Low classes (value = −0.77) in the relevance network. Three OTUs had a positive link with the Low class. Table 2 shows least square means for each t10t11R class for discriminant genera and OTUs and their affiliation groups. OTU52 and OTU21 had very low abundances in the Low class, and 209 and 151 times higher abundances in the High class, respectively. OTUs 21, 32 and 52, that positively linked with high t10t11R represented only a minor part of their affiliation group except in the High class, and these affiliation groups had no significant relationship with the t10t11R. OTUs 984 and 64 were affiliated at species (*Olsenella scatoligenes*) and genus (*Olsenella*) level, respectively, and also positively linked with the t10t11R. Two out of the three OTUs (15 and 16) that were negatively linked with the t10t11R were affiliated to *Rikenellaceae RC9 gut group*, whose abundance was lower in the High than the Low class.

**Table 2:** Relative abundance (least squared mean percentages ± SE of GBM transformed values) of genera and OTUs selected by sPLS-DA (bold) and their affiliation groups for the three classes of *trans*-10/*trans*-11 ratio.

	Low	Medium	High	*p*
*Proteobacteria*	3.35 ± 2.59	8.42 ± 2.39	11.66 ± 2.66	0.181
*Succinivibrionaceae*	2.92 ± 2.67	7.94 ± 2.45	11.1 ± 2.74	0.456
***Succinivibrionaceae UCG-001***	**0.84**^**a**^ **± 2.76**	**5.83**^**b**^ **± 2.45**	**10.34**^**b**^ **± 2.79**	**0.000**
*Actinobacteriota*	2.11 ± 0.82	1.50 ± 1.10	2.27 ± 1.03	0.052
*Atopobiaceae*	0.34^a^ ± 0.17	0.74^b^ ± 0.22	1.83^c^ ± 0.21	0.000
***Olsenella***	**0.19**^**a**^ **± 0.16**	**0.44**^**b**^ **± 0.21**	**1.74**^**c**^ **± 0.2**	**0.000**
***OTU64***	**0.04**^**a**^ **± 0.14**	**0.14**^**b**^ **± 0.19**	**1.03**^**c**^ **± 0.17**	**0.000**
***O. scatolinogenes OTU984***	**0.02**^**a**^ **± 0.03**	**0.11**^**b**^ **± 0.03**	**0.19**^**c**^ **± 0.03**	**0.000**
*Bacteroidota*	56.59 ± 2.79	57.16 ± 3.74	50.57 ± 3.49	0.511
*Rikenellaceae*	6.50^b^ ± 0.79	3.34^ab^ ± 0.94	2.22^a^ ± 0.92	0.010
*Rikenellaceae RC9 gut group*	6.31^b^ ± 0.77	3.18^ab^ ± 0.92	2.15^a^ ± 0.9	0.009
***OTU15***	**1.00**^**c**^ **± 0.17**	**0.32**^**b**^ **± 0.21**	**0.07**^**a**^ **± 0.21**	**0.000**
***OTU16***	**0.66**^**b**^ **± 0.14**	**0.04**^**a**^ **± 0.18**	**0.03**^**a**^ **± 0.17**	**0.000**
*Muribaculaceae*	4.98 ± 1.51	11.41 ± 2.03	13.34 ± 1.89	0.075
***OTU21***	**0.02**^**a**^ **± 0.6**	**0.12**^**a**^ **± 0.8**	**3.02**^**b**^ **± 0.75**	**0.000**
*p-251-o5*	1.14^b^ ± 0.22	0.21^ab^ ± 0.28	0.16^a^ ± 0.27	0.006
***OTU25***	**0.79**^**b**^ **± 0.16**	**0.08**^**a**^ **± 0.2**	**0.05**^**a**^ **± 0.19**	**0.000**
*Firmicutes*	36.55 ± 2.65	32.4^0^ ± 3.55	34.30 ± 3.32	0.607
*[Eubacterium] coprostanoligenes group*	1.53 ± 0.61	0.89 ± 0.82	2.40 ± 0.76	0.128
***OTU52***	**0.01**^**a**^ **± 0.12**	**0.05**^**a**^ **± 0.16**	**2.09**^**b**^ **± 0.15**	**0.000**
*Lachnospiraceae*	12.06^a^ ± 1.59	15.95^ab^ ± 2.06	20.10^b^ ± 1.94	0.000
*Lachnospiraceae NK3A20 group*	3.68 ± 0.72	4.10 ± 0.94	4.65 ± 0.88	0.286
***OTU32***	**0.22**^**a**^ **± 0.17**	**0.59**^**b**^ **± 0.22**	**1.45**^**c**^ **± 0.21**	**0.000**
***[Eubacterium] ruminantium group***	**0.37**^**a**^ **± 0.2**	**0.39**^**ab**^ **± 0.27**	**1.04**^**b**^ **± 0.25**	**0.006**
***Shuttleworthia***	**0.24**^**a**^ **± 0.29**	**0.32**^**a**^ **± 0.39**	**1.68**^**b**^ **± 0.36**	**0.000**
***[Ruminococcus] gauvreauii group***	**0.53**^**a**^ **± 0.26**	**1.43**^**b**^ **± 0.34**	**2.48**^**b**^ **± 0.32**	**0.000**
***Syntrophococcus***	**0.24**^**a**^ **± 0.19**	**0.42**^**a**^ **± 0.26**	**1.78**^**b**^ **± 0.24**	**0.000**
*Oscillospiraceae*	4.56 ± 0.45	2.59 ± 0.60	1.69 ± 0.56	0.074
***Papillibacter***	**0.26**^**b**^ **± 0.06**	**0.04**^**a**^ **± 0.08**	**0.03**^**a**^ **± 0.08**	**0.000**
*Eubacteriaceae*	0.01^a^ ± 0.02	0.05^b^ ± 0.02	0.12^b^ ± 0.02	0.000
***Pseudoramibacter***	**0.01**^**a**^ **± 0.02**	**0.05**^**b**^ **± 0.02**	**0.12**^**b**^ **± 0.02**	**0.000**
*Veillonellaceae*	0.07^a^ ± 0.22	0.36^a^ ± 0.29	1.74^b^ ± 0.27	0.000
***Dialister***	**0.07**^**a**^ **± 0.22**	**0.36**^**a**^ **± 0.29**	**1.74**^**b**^ **± 0.27**	**0.000**
*Anaerovoracaceae*	1.42 ± 0.16	0.94 ± 0.21	1.28 ± 0.19	0.344
***[Eubacterium] nodatum group***	**0.07**^**a**^ **± 0.06**	**0.19**^**b**^ **± 0.07**	**0.56**^**b**^ **± 0.07**	**0.000**

## Discussion

### Biohydrogenation pathways

CLA are the first intermediates of a series of reactions affecting LA, with stearic acid as a final product. The first reaction is an isomerization of LA whose one of the double bonds is displaced and its geometric configuration is changed from *cis* to *trans*. Usually, this isomerization affects the Δ12 double-bond leading to c9t11 CLA. *B. fibrisolvens* and *B. hungatei* are the best known rumen biohydrogenating bacteria. They isomerize LA to mainly c9t11 CLA and reduce it to t11 C18:1. *B. proteoclasticus* can also perform these two reactions and is able to reduce t11 C18:1 to stearic acid. In MFD conditions associated with the *trans*-10 BH shift, the isomerization mainly affects the Δ9 double bond, leading to t10c12 CLA, but involved microorganisms are still controversial. This isomer can in turn be reduced to t10C18:1 by *B. fibrisolvens* and *B. hungatei* and to stearic acid by *B. proteoclasticus*. Only t10c12 CLA has been unequivocally shown to affect mammary metabolism (Baumgard et al., 2001), reducing circulating FA uptake and *de novo* FA synthesis from acetate and butyrate absorbed at the rumen level, and incorporation of FA into triacylglycerols (Baumgard et al., 2002). However, other minor isomers could be involved (Leskinen et al., 2019). The isomerization and the first reduction are rapid reactions, but the last reduction is slower, so that rumen CLA concentrations are much lower than *trans*-C18:1 concentrations. As a result, the use of the sum of t10 intermediates is analytically more reliable than t10c12 CLA alone. Moreover, rumen percentage of total *trans* FA relative to total FA depends on diet and time relative to meal, so that the t10t11R is a better proxy than the t10 percentage to characterize the t10-shift. There is no scientific consensus about a threshold for the different proxys of t10-shift. Recently, Andreen et al. (2021) considered cows having more than 0.6% t10 C18:1 in total milk FA as experiencing MFD. In our field dataset, this corresponded with a 0.45 t10t11R. A 1.0 t10t11R threshold to characterize the t10-shift has been mentioned by Toral et al. (2020). Our 0.35 et 1.0 thresholds of t10t11R are consistent with these values.

The t10t11R range was much higher in the induced than the field dataset. Previous studies inducing MFD in lactating dairy cows also resulted in milk t10t11R that were around 5.0 on average with MFD inducing diets (Rico and Harvatine, 2013; Fougère et al., 2018; Leskinen et al., 2019) and were associated with milk fat contents under 25 g/kg. The 7.97 t10t11R observed in our induction study on dry dairy cows and in published studies on lactating dairy cows makes it possible to consider that our rumen conditions could be studied together with data obtained on lactating cows in our field study. In our field dataset, the average milk t10t11R in the High class was 1.86, and was associated with a 22.6 g/kg milk fat content. Interestingly, as previously reported by Conte et al. (2018) in a field study, cows receiving the same diet exhibited a wide range of t10t11R, suggesting strong individual susceptibility to the t10-shift. Besides, as ours, this study showed that milk production is not associated with this ratio (*p* = 0.645 between non-MFD and MFD cows in the study of Conte et al. (2018), *r* = −0.28 and *p* = 0.201 in our study). Our observed relationship between t10t11R and milk fat content fitted with previous observations showing curvilinear relationship between milk t10 FA and milk fat content (Matamoros et al., 2020).

### Bacterial community: Diversity and changes at phylum level

Known biohydrogenating rumen bacteria isomerize either *via* the t10 or *via* the t11 pathway, which means that the t10-shift necessarily relates to changes of abundances or activities of these bacteria. However, previous studies (Weimer et al., 2010; Pitta et al., 2018) evidenced much wider ruminal microbiota changes associated with MFD than changes limited to known biohydrogenating bacteria. In our study, richness and alpha diversity indexes were lower in Medium and High classes compared to the Low class. This indicates a predominance of some taxa in high t10 producing rumen samples, and is in accordance with increased observed species and Shannon index during recovery from MFD (Pitta et al., 2018) or MFD alleviation by 2-hydroxy-4-(methylthio) butanoate supplementation (Pitta et al., 2020). The reduction of richness was also evidenced by the much lower number of unique OTUs in the High and Medium classes compared to the Low class (1, 3 and 162, respectively). Similar changes of richness and diversity have also been reported in cows with subacute ruminal acidosis, and associated with lower adaptability, functionality and robustness of the microbiota (Plaizier et al., 2017a).

At phylum level, only *Desulfobacterota* and *Proteobacteria* were linked with t10t11R, with higher abundances observed in the High class. MFD is often associated with subacute ruminal acidosis, and t10 FA have been used as a proxy to characterize acidosis (Enjalbert et al., 2008; Jing et al., 2018). Induced subacute ruminal acidosis results in increased abundance of *Firmicutes* (Plaizier et al., 2017a) and decreased abundance of *Bacteroidetes*, *Proteobacteria*, *Spirochetes* and *Actinobacteria* (Mao et al., 2013), but this link between *Firmicutes*:*Bacteroidetes* ratio and acidosis is not always observed (Callaway et al., 2010). Also differing from our study, Jami et al. (2014), comparing microbiota between cows receiving the same diet, reported that the *Firmicutes*:*Bacteroidetes* ratio is positively correlated with milk fat yield. However, they used a diet with 70% concentrate, which strongly differed from our conditions.

### Bacteria associated with the *trans*-10 biohydrogenation pathway

**Figure. 4:**
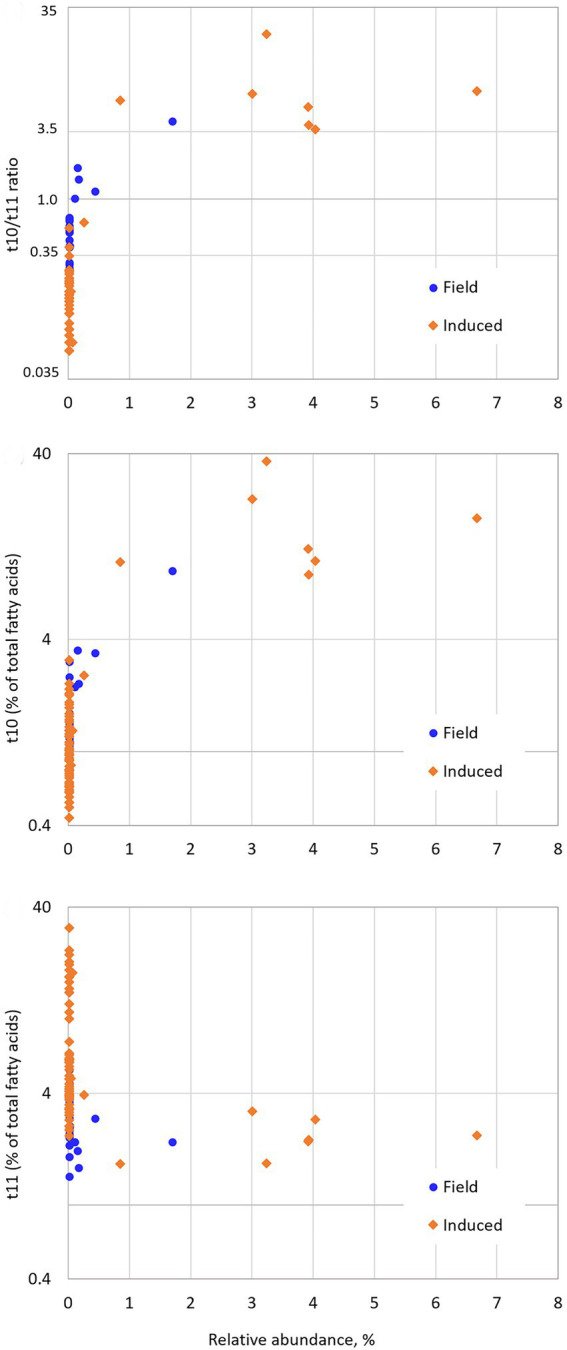
Scatterplots of the relationship between rumen *trans*-10/*trans*-11 ratio, *trans*-10 an *trans*-11 fatty acids percentages and GBM-transformed relative abundance of OTU52.

Sparse PLS-DA evidenced that the most strongly positively associated OTU with the High t10t11R class was OTU52, belonging to *[Eubacterium] coprostanoligene*s *group*, at family level. Indeed, this OTU had a 27.8 VIP on component 1, and a 0.015% relative abundance in the Low class as opposed to 2.1% in the High class (*p* < 0.001) on average, but its relative abundance in the Medium class did not differ from that in the Low class (Table 2). In our datasets, its high abundances were always associated with high t10t11R due to both high t10 FA and low t11 FA percentages (Figure 4). At the whole family level, relative abundance of *[Eubacterium] coprostanoligenes group* did not differ between classes, and this group has not been evidenced in previous studies relative to the t10-shift. OTU52 represented 86% of *[Eubacterium] coprostanoligenes group* abundance in the High class, as opposed to 1% in the Low class, which suggests that different strains within this group were differently affected by dietary conditions and differently associated with the t10-shift. *E. coprostanoligenes* is a cholesterol-reducing bacteria. Ruminant’s diet does not contain cholesterol, but the strain studied by Freier et al. (1994) does not require cholesterol for growth which makes its presence in the rumen possible, whereas its functions remain unknown in this digestive compartment. Consistent with our data, the relative abundance of *[Eubacterium] coprostanoligenes* in the rumen has been reported to be around 1% in the rumen and it is identified in more 93.5% of rumen samples (Holman and Gzyl, 2019). Its abundance is slightly but significantly decreased by linseed oil plus nitrate supplementation (Popova et al., 2017), strongly decreased by rumen by-pass fat (Liu et al., 2019), and is higher in high-yield than low-yield cows receiving the same diet (Tong et al., 2018). Microbial reduction of cholesterol to coprostanol involves four successive reactions: an initial oxidation followed by an isomerization changing the double-bond position, and two reductions (Ren et al., 1996). Liavonchanka et al. (2009) proposed a mechanism involving a transient oxidation of LA for isomerization to t10c12 CLA. Hence, the oxidation and isomerization abilities of *[Eubacterium] coprostanoligenes* or specific strains of this group could be associated with LA isomerization *via* the *trans*-10 pathway. However, both mechanisms of LA isomerization *via* the t10 pathway and cholesterol oxidation are not sufficiently known to clearly draw a possible involvement of *E. coprostanoligenes* in t10 isomerization of LA in the rumen. A possible implication of FA oxidation reactions in MFD is also suggested by the results of Ventto et al. (2017) who showed that 10-oxo C18:0 was strongly increased when soybean oil was added to a high concentrate diet inducing a MFD. Toral et al. (2016) hypothesised a putative involvement of *Quinella ovalis* and unclassified *Veillonellaceae* in these oxidation processes. *Quinella* was not found in our dataset, but the *Veillonellaceae* family was more abundant in the High than in the Medium and Low classes.

Similarly, OTU21 belonging to *Muribaculaceae* had a 11.3 VIP and a much higher abundance in the High than the Low and Medium classes. This OTU represented only a minor part of its family, which has already been isolated in the rumen, with changes of abundance associated with dietary transition at weaning in goat kids (Guo et al., 2020) or dietary change after calving in dairy cows (Bach et al., 2018). In rodents, the abundance of *Muribaculaceae* increases with high fat diets (Gérard, 2020), but no function of this family on FA metabolism has been described (Lagkouvardos et al., 2019) and no association between these bacteria and the t10-shift has been evidenced in previous studies.

OTU32, belonging to the *Lachnospiraceae NK3A20 group*, was also associated with the High class. This group has 89% similarity with two strains of *Butyrivibrio*, including one known strain of *B. proteoclasticus* (Kenters et al., 2011), known to biohydrogenate *via* the t11 pathway, which is not consistent with our observed positive link with t10t11R.

Two OTUs belonging to *Olsenella* genus were also found discriminant between t10t11R classes, whereas with lower VIPs than OTU52 and OTU21. At genus level, *Olsenella* was also among selected discriminant genera and was positively associated with t10t11R. This genus has not been reported in other t10-shift studies, but the *Coriobacteriaceae* family positively correlated with the t10-shift in previous experiments (Dewanckele et al., 2018; Pitta et al., 2018, 2020). Although *Olsenella* is now affiliated to the *Atopobiaceae* family, it was affiliated to the *Coriobacteriaceae* in the GreenGenes databases used by these authors. *Olsenella* has already been shown to have a higher abundance with high grain compared to hay diets (Kim et al., 2018) and with fat supplemented diets (Kim et al., 2014). In our dataset, OTU984 was the only OTU affiliated to *O. scatoligenes* species. It represented a minor part of *Olsenella* genus and was also positively associated with t10t11R. This species has already been found in rumen samples^1^ but had not previously been described in MFD or t10-shift studies. Its possible functions in BH remain unknown. The *Syntrophococcus* genus was also positively associated with the t10t11R and is also known to have a higher abundance with fat supplemented diets in cattle (Kim et al., 2014). *S. sucromutans* requires C18:1 FA for growth but this species is not involved in BH (Doré and Bryant, 1989).

**Figure 5:**
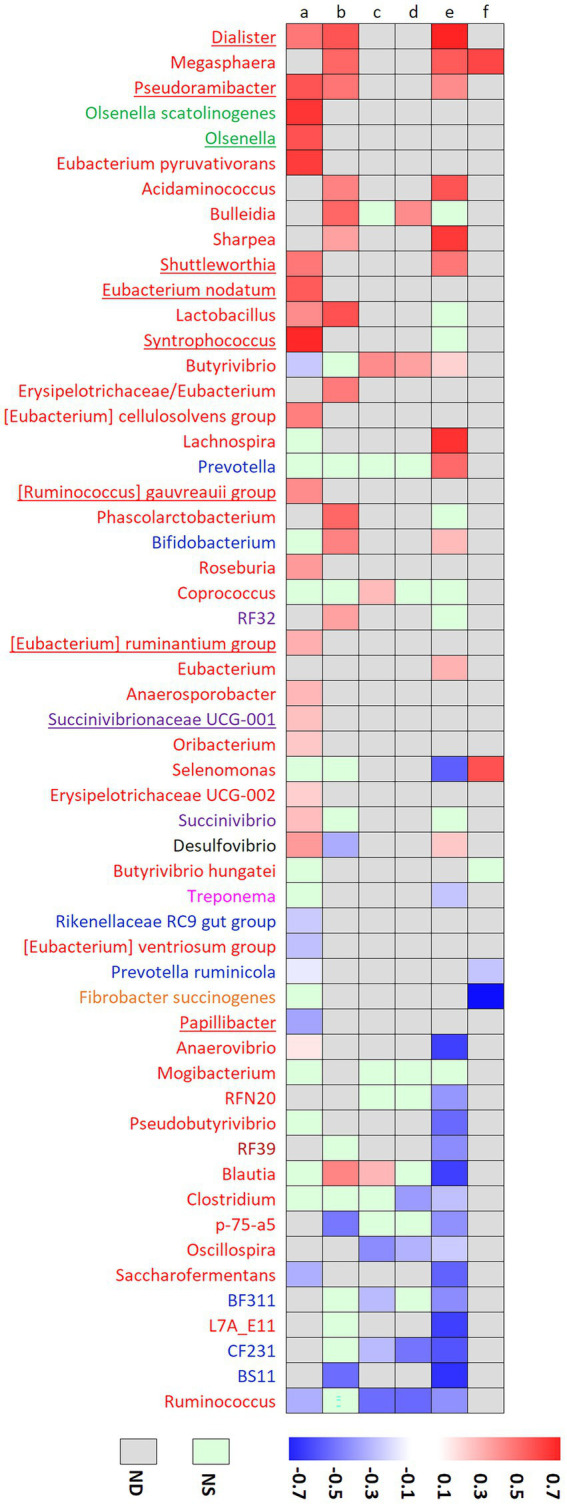
Correlation coefficients between genera or species and rumen trans-10 / trans-11 ratio in our experiment (a) and the experiment of Dewanckele et al. (2019) (b), or with trans-10 isomers percentage in the liquid (c) or solid (d) phases of the rumen in the experiment of Pitta et al. (2018), or with milk percentages of trans-10 C18:1 in the experiments of Pitta et al. (2020) (e) and Rico et al. (2015) (f). Only taxa analyzed in our study or at least two studies were selected. Color name coding represents the phylum to which each genus belongs: *Actinobacteria* (green), *Bacteroidetes* (blue), *Desulfobacterota* (black), *Fibrobacteres* (orange), *Firmicutes* (red), *Proteobacteria* (purple), *Spirochaetae* (pink) and *Tenericutes* (brown). sPLS-DA discriminant genera are underlined.

Biohydrogenation *via* the t10 pathway has also been explored in culture studies using bacterial strains isolated from the rumen. *Megasphaera elsdenii* (Kim et al., 2002), *Cutibacterium acnes* (formerly *Propionibacterium acne*s) (Wallace et al., 2007), *Bifidobacterium pseudolongum* and *B. thermophilum* (Jaglan et al., 2019) have been shown to be able to synthesize t10c12CLA from LA, but their implication is somewhat controversial. Figure 5 reports correlations between genera or species relative abundances and biomarkers of t10-shift (t10t11R or t10 isomers percentage in rumen or milk) in our experiment and published literature based on t10-shift experimental induction. Correlation was strongly positive for *Megasphaera* genus or *M. elsdenii* in three experiments where t10-shift was induced using C22:6 n-3 on goats (Dewanckele et al., 2018) or a high grain – high fat diet on cows (Rico et al., 2015; Pitta et al., 2020). High grain diets are known to increase the abundance of *M. elsdenii* (Plaizier et al., 2017b) so that the high abundance of this species could have been a consequence of the induction diet but not necessarily a cause of the t10-shift. However, *Megasphera* genus was not found in our datasets, including in the induction study. *Bifidobacterium* genus was positively correlated with t10 FA in previous experiments (Dewanckele et al., 2018; Pitta et al., 2020), but the correlation was not significant in our datasets. Similar to our results, *C. acnes* has not been reported in previous studies.

In our study, *Dialister* and *Pseudoramibacter* positively correlated with the t10t11R without being among the most discriminant genera. They have already been shown to positively correlate with t10-shift markers (Figure 5). On the contrary, *Eubacterium nodatum*, [*Eubacterium*] *ruminantium group*, [*Ruminococcus*] *gauvrei group*, *Shuttleworthia*, *Synthrophococcus* and *Succinovibrionaceae UCG-001* have not been yet reported to be positively linked with the t10-shift. Some strains of *Roseburia* and *Eubacterium ruminantium*, that positively correlated with t10t11R in our study, can metabolize LA but production of t10 FA was not reported (Devillard et al., 2007).

### Bacteria associated with the *trans*-11 biohydrogenation pathway

Regarding the t11 pathway of ruminal BH, *Butyrivibrio* is the most studied genus. In our study, the relative abundance of this genus was significantly lower in the High than in the Low class (0.23 and 0.85%, respectively), the Medium class having an intermediate and non-significantly different abundance. Only one OTU could be affiliated at species level (*B. hungatei*) and its abundance did not differ among t10t11R classes. Enjalbert et al. (2017) hypothesized that the t10-shift could be due to a lower capacity of ruminal bacteria, including *Butyrivibrio*, to isomerize LA when they are inhibited by starch or oil addition. This is consistent with the present study or that of Rico et al. (2015) who showed a negative correlation between *B. fibrisolvens*/*Pseudobutyrivibrio* group and t10 C18:1. However, others showed a positive link between *Butyrivibrio* abundances and t10 isomers (Figure 5). This lack of consistency can reflect a lack of relationship between abundance and activity, especially for BH which is a detoxification and not a nutritional process. Abundance of biohydrogenating bacteria is probably more strongly linked to their ability to grow on a carbohydrate substrate than to their ability to hydrogenate unsaturated FA (Enjalbert et al., 2017). *Sharpea*, another t11 producing bacteria (Dewanckele et al., 2019) was not found in our datasets, and, as opposed to this known function, was positively linked with t10-shift markers in previous experiments.

In our study, three OTUs, two of them belonging to *Rikenellaceae* RC9 gut group (OTU15 and OTU16) and one to the p-251-o5 family (OTU25), discriminated for the Low class, and their relative abundances differed between t10t11R classes. The *Papillibacter* genus was discriminant and associated with low t10t11R. Its abundance is known to decrease when increasing grain (Mao et al., 2013) or fat (Huws et al., 2014), which are conditions that favour the t10-shift. However, ruminal functions of *Rikenellaceae RC9 gut group*, *p-251-o5* and *Papillibacter* are largely unknown.

### OTUs and genera as markers of biohydrogenation changes

**Figure 6:**
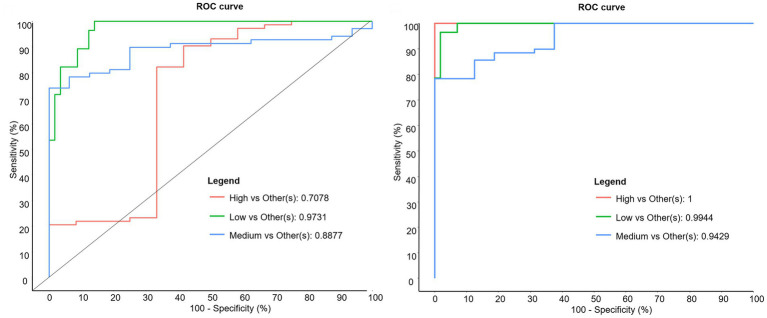
Receiving operator characteristic curves discriminating between *trans*-10/*trans*-11 ratio classes at genus (left) and OTU (right) levels.

That most discriminant genera identified by sPLS-DA are not clearly involved in the ruminal BH process suggests that ruminal disturbances associated with the t10-shift go far beyond changes of BH bacteria, and that the shift of BH and associated MFD could be only part of changes due to high starch or high unsaturated FA diets. However, genera and OTUs identified by sPLS-DA can be considered as markers of ruminal microbiota biohydrogenating either by the t11 or the t10 pathway. Receiver operating characteristic curves calculated from sPLS-DA showed that OTU level provides a much better sensitivity and specificity to distinguish between the High t10t11R class and others than genus level (Figure 6), consistent with known strain specificity within bacterial species for ruminal BH (Hussain et al., 2016).

## Conclusion

Based on a shift of rumen function relative to unsaturated FA BH, and associating data from an experimental induction study and a spontaneous field observation, we showed that analysis of ruminal bacterial community at OTU level revealed a much better relationship between biochemical and taxonomic changes than genus level, which is consistent with the known within genus or within species strain specificity. Changes of a low number of OTUs can represent the molecular signature of functional changes, and, although a mathematical link between abundance of a bacteria and t10-shift cannot be univocally interpreted as a cause-effect relationship, some discriminant OTUs can be considered as candidates for culture studies aiming at identifying bacteria responsible of this BH shift.

## Data availability statement

The datasets presented in this study can be found in online repositories. The names of the repository/repositories and accession number(s) can be found at: https://www.ncbi.nlm.nih.gov/, PRJNA735057.

## Ethics statement

Ethical review and approval was not required for the animal study because the experimental study began before approvement by Ethic Committee became mandatory in France. All procedures complied with the Guide for the Care and Use of Agricultural Animals in Research and Teaching, Federation of Animal Science Societies, 2010. Written informed consent for participation was not obtained from the owners because a written consent was not mandatory at this time.

## Author contributions

FE and AZ conceived the experiments and participated in sample collection and processing. LC and FE performed data processing and statistical analyses. FE, AZ, and AM participated in the interpretation of the results. FE and AM wrote the manuscript. All authors contributed to the article and approved the submitted version.

## Funding

The experiment was funded by GenPhySE, Université de Toulouse, INRAE, ENVT, INPT, Toulouse, France.

## Conflict of interest

The authors declare that the research was conducted in the absence of any commercial or financial relationships that could be construed as a potential conflict of interest.

## Publisher’s note

All claims expressed in this article are solely those of the authors and do not necessarily represent those of their affiliated organizations, or those of the publisher, the editors and the reviewers. Any product that may be evaluated in this article, or claim that may be made by its manufacturer, is not guaranteed or endorsed by the publisher.

## Footnotes

^1^ https://www.arb-silva.de/browser/ssu-138.1/LOJF01000014

## References

[ref1] Andreen D. M. Haan M. M. Dechow C. D. Harvatine K. J. (2021). Determination of relationships between rumination and milk fat concentration and fatty acid profile using data from commercial rumination sensing systems. J. Dairy Sci. 104, 8901–8917. doi: 10.3168/jds.2020-19860, PMID: 34024599

[ref2] Arbonés-Mainar J. M. Navarro M. A. Guzmáan M. A. Arnal C. Surra J. C. Acín S.. (2006). Selective effect of conjugated linoleic acid isomers on atherosclerotic lesion development in apolipoprotein E knockout mice. Atherosclerosis 189, 318–327. doi: 10.1016/j.atherosclerosis.2006.01.015, PMID: 16530768

[ref3] Bach A. López-García A. González-Recio O. Elcoso G. Fàbregas F. Chaucheyras-Durand F.. (2018). Changes in the rumen and colon microbiota and effects of live yeast dietary supplementation during the transition from the dry period to lactation of dairy cows. J. Dairy Sci. 102, 6180–6198. doi: 10.3168/jds.2018-16105, PMID: 31056321

[ref4] Baumgard L. H. Matitashvili E. Corl B. A. Dwyer D. A. Bauman D. E. (2002). *Trans*-10, *cis*-12 conjugated linoleic acid decreases lipogenic rates and expression of genes involved in milk lipid synthesis in dairy cows. J. Dairy Sci. 85, 2155–2163. doi: 10.3168/jds.S0022-0302(02)74294-X, PMID: 12362447

[ref5] Baumgard L. H. Sangster J. K. Bauman D. E. (2001). Milk fat synthesis in dairy cows is progressively reduced by increasing supplemental amounts of *trans*-10, *cis*-12 conjugated linoleic acid (CLA). J. Nutr. 131, 1764–1769. doi: 10.1093/jn/131.6.1764, PMID: 11385065

[ref6] Bokulich N. A. Subramanian S. Faith J. J. Gevers D. Gordon J. I. Knight R.. (2013). Quality-filtering vastly improves diversity estimates from Illumina amplicon sequencing. Nat. Methods 10, 57–59. doi: 10.1038/nmeth.2276, PMID: 23202435 PMC3531572

[ref7] Callaway T. R. Dowd S. E. Edrington T. S. Anderson R. C. Krueger N. Bauer N.. (2010). Evaluation of bacterial diversity in the rumen and feces of cattle fed different levels of dried distillers grains plus solubles using bacterial tag-encoded FLX amplicon pyrosequencing. J. Anim. Sci. 88, 3977–3983. doi: 10.2527/jas.2010-2900, PMID: 20729286

[ref8] Conte G. Dimauro C. Serra A. Macciotta N. P. P. Mele M. (2018). A canonical discriminant analysis to study the association between milk fatty acids of ruminal origin and milk fat depression in dairy cows. J. Dairy Sci. 101, 6497–6510. doi: 10.3168/jds.2017-13941, PMID: 29627248

[ref9] Devillard E. McIntosh F. M. Duncan S. H. Wallace R. J. (2007). Metabolism of linoleic acid by human gut bacteria: different routes for biosynthesis of conjugated linoleic acid. J. Bacteriol. 189, 2566–2570. doi: 10.1128/JB.01359-06, PMID: 17209019 PMC1899373

[ref10] Dewanckele L. Jeyanathan J. Vlaeminck B. Fievez V. (2020). Identifying and exploring biohydrogenating rumen bacteria with emphasis on pathways including *trans*-10 intermediates. BMC Microbiol. 20:198. doi: 10.1186/s12866-020-01876-7, PMID: 32635901 PMC7339423

[ref11] Dewanckele L. Vlaeminck B. Fievez V. (2019). *Sharpea azabuensis*: a ruminal bacterium that produces *trans*-11 intermediates from linoleic and linolenic acid. Microbiology 165, 772–778. doi: 10.1099/mic.0.000811, PMID: 31100055

[ref12] Dewanckele L. Vlaeminck B. Hernandez-Sanabria E. Ruiz-González A. Debruyne S. Jeyanathan J.. (2018). Rumen biohydrogenation and microbial community changes upon early life supplementation of 22:6n-3 enriched microalgae to goats. Front. Microbiol. 9:573. doi: 10.3389/fmicb.2018.00573, PMID: 29636742 PMC5880937

[ref13] Doré J. Bryant P. (1989). Lipid growth requirement and influence of lipid supplement on fatty acid and aldehyde composition of *Syntrophococcus sucromutans*. Appl. Environ. Microbiol. 55, 927–933. doi: 10.1128/aem.55.4.927-933.1989, PMID: 2729991 PMC184226

[ref14] Enjalbert F. Combes S. Zened A. Meynadier A. (2017). Rumen microbiota and dietary fat: a mutual shaping. J. Appl. Microbiol. 123, 782–797. doi: 10.1111/jam.13501, PMID: 28557277

[ref15] Enjalbert F. Videau Y. Nicot M.-C. Troegeler-Meynadier A. (2008). Effects of induced subacute ruminal acidosis on milk fat content and milk fatty acid profile. J. Anim. Physiol. Anim. Nutr. 92, 284–291. doi: 10.1111/j.1439-0396.2007.00765.x, PMID: 18477308

[ref16] Escudié F. Auer L. Bernard M. Mariadassou M. Cauquil L. Vidal K.. (2018). FROGS: find. Rapidly. OTUs with galaxy solution. Bioinformatics 34, 1287–1294. doi: 10.1093/bioinformatics/btx791, PMID: 29228191

[ref17] Fougère H. Delavaud C. Bernard L. (2018). Diets supplemented with starch and corn oil, marine algae, or hydrogenated palm oil differentially modulate milk fat secretion and composition in cows and goats: a comparative study. J. Dairy Sci. 101, 8429–8445. doi: 10.3168/jds.2018-14483, PMID: 29885893

[ref18] Freier T. A. Beitz D. C. Hatman P. (1994). Characterization of *Eubacterium coprostanoligenes* sp. nov, a cholesterol-reducing anaerobe. Int. J. System. Bacteriol. 44, 137–142. doi: 10.1099/00207713-44-1-137, PMID: 8123557

[ref19] Gérard P. (2020). The crosstalk between the gut microbiota and lipids. Oilseeds Fats Crops Lipids 27:70. doi: 10.1051/ocl/2020070

[ref20] González I. Lê Cao K. A. Dabis M. J. Dejean S. (2012). Visualising associations between paired ‘omics’ data sets. Bio Data Mining 5, 19–23. doi: 10.1186/1756-0381-5-19, PMID: 23148523 PMC3630015

[ref21] Griinari J. M. Dwyer D. A. Mcguire M. A. Bauman D. E. Palmquist D. L. Nurmela K. V. V. (1998). *Trans*-octadecenoic acids and milk fat depression in lactating dairy cows. J. Dairy Sci. 81, 1251–1261. doi: 10.3168/jds.S0022-0302(98)75686-3, PMID: 9621226

[ref22] Guo J. Li P. Zhang K. Zhang L. Wang X. Li L.. (2020). Distinct stage changes in early-life colonization and acquisition of the gut microbiota and its correlations with volatile fatty acids in goat kids. Front. Microbiol. 11:584742. doi: 10.3389/fmicb.2020.584742, PMID: 33162961 PMC7581860

[ref23] Holman D. B. Gzyl K. E. (2019). A meta-analysis of the bovine gastrointestinal tract microbiota. FEMS Microbiol. Ecol. 95:072. doi: 10.1093/femsec/fiz072 31116403

[ref24] Hussain S. K. A. Sivastava A. Tyagi A. Lumar Shandilya U. Kumar A. Kumar S.. (2016). Characterization of CLA-producing *Butyrivibrio* spp. reveals strain-specific variations. 3. Biotech 6:90. doi: 10.1007/s13205-016-0401-2 PMC4786556 28330160

[ref25] Huws S. A. Kim E. J. Cameron S. J. S. Girdwood S. E. Davies L. Tweed J.. (2014). Characterization of the rumen lipidome and microbiome of steers fed a diet supplemented with flax and echium oil. Microb. Biotech. 8, 331–341. doi: 10.1111/1751-7915.12164 PMC4353346 25223749

[ref26] Jaglan N. Kumar S. Choudhury P. K. Tyagi B. Tyagi A. K. (2019). Isolation, characterization and conjugated linoleic acid production potential of bifidobacterial isolates from ruminal fluid samples of Murrah buffaloes. Anaerobe 56, 40–45. doi: 10.1016/j.anaerobe.2019.02.001, PMID: 30738138

[ref27] Jami E. White B. A. Mizrahi I. (2014). Potential role of the bovine rumen microbiome in modulating milk composition and feed efficiency. PloS ONE 9:e85423. doi: 10.1371/journal.pone.0085423, PMID: 24465556 PMC3899005

[ref28] Jing L. Dewanckele L. Vlaeminck B. Van Straalen W. M. Koopmans A. Fievez V. (2018). Susceptibility of dairy cows to subacute ruminal acidosis is reflected in milk fatty acid proportions, with C18:1 *trans*-10 as primary and C15: 0 and C18:1 *trans*-11 as secondary indicators. J. Dairy Sci. 101, 9827–9840. doi: 10.3168/jds.2018-14903, PMID: 30172392

[ref29] Kenters N. Henderson G. Jeyanathan J. Kittelmann S. Janssen P. H. (2011). Isolation of previously uncultured rumen bacteria by dilution to extinction using a new liquid culture medium. J. Microbiol. Methods 84, 52–60. doi: 10.1016/j.mimet.2010.10.011, PMID: 21034781

[ref30] Kim M. Eastridge M. L. Yu Z. (2014). Investigation of ruminal bacterial diversity in dairy cattle fed supplementary monensin alone and in combination with fat, using pyrosequencing analysis. Can. J. Microbiol. 60, 65–71. doi: 10.1139/cjm-2013-0746, PMID: 24498983

[ref31] Kim Y. J. Liu R. H. Rychlik J. L. Russell J. B. (2002). The enrichment of a ruminal bacterium (Megasphaera elsdenii YJ-4) that produces the *trans*-10, *cis*-12 isomer of conjugated linoleic acid. J. Appl. Microbiol. 92, 976–982. doi: 10.1046/j.1365-2672.2002.01610.x, PMID: 11972704

[ref32] Kim Y. H. Nagata R. Ohkubo A. Ohtani N. Kushibiki S. Ichijo T.. (2018). Changes in ruminal and reticular pH and bacterial communities in Holstein cattle fed a high-grain diet. BMC Vet. Res. 14:310. doi: 10.1186/s12917-018-1637-3, PMID: 30314483 PMC6186129

[ref33] Lagkouvardos I. Lesker T. R. Hitch T. C. A. Gálvez E. J. C. Smit N. Neuhaus K.. (2019). Sequence and cultivation study of *Muribaculaceae* reveals novel species, host preference, and functional potential of this yet undescribed family. Microbiome 7:28. doi: 10.1186/s40168-019-0637-2, PMID: 30782206 PMC6381624

[ref34] Lee C. Copelin J. E. Park T. Mitchell K. E. Firkins J. L. Socha M. T.. (2021). Effects of diet fermentability and supplementation of 2-hydroxy-4-(methylthio)-butanoic acid and isoacids on milk fat depression: 2. Ruminal fermentation, fatty acid, and bacterial community structure. J. Dairy Sci. 104, 1604–1619. doi: 10.3168/jds.2020-18950, PMID: 33358812

[ref35] Leskinen L. Ventto L. Kairenius P. Shingfield K. J. Vilkki J. (2019). Temporal changes in milk fatty acid composition during diet-induced milk fat depression in lactating cows. J. Dairy Sci. 102, 5148–5160. doi: 10.3168/jds.2018-15860, PMID: 30904304

[ref36] Liavonchanka A. Rudolph M. G. Tittmann K. Hamberg M. Feussner I. (2009). On the mechanism of a polyunsaturated fatty acid double bond isomerase from *Propionibacterium acnes*. J. Biol. Chem. 284, 8005–8012. doi: 10.1074/jbc.M809060200, PMID: 19164287 PMC2658094

[ref37] Liu Y. Z. Chen S. Zhao W. Lang M. Zhang X. F. Wang T.. (2019). Effects of yeast culture supplementation and the ratio of non-structural carbohydrate to fat on rumen fermentation parameters and bacterial-community composition in sheep. Anim. Feed Sci. Technol. 249, 62–75. doi: 10.1016/j.anifeedsci.2019.02.003

[ref38] Maia M. R. G. Chaudhary L. C. Figueres L. Wallace R. J. (2007). Metabolism of polyunsaturated fatty acids and their toxicity to the microflora of the rumen. Antonie Van Leeuwenhoek 91, 303–314. doi: 10.1007/s10482-006-9118-2, PMID: 17072533

[ref39] Mao S. Y. Zhang R. Y. Wang D. S. Zhu W.-Y. (2013). Impact of subacute ruminal acidosis (SARA) adaptation on rumen microbiota in dairy cattle using pyrosequencing. Anaerobe 24, 12–19. doi: 10.1016/j.anaerobe.2013.08.003, PMID: 23994204

[ref40] Martín-Fernández J. A. Hron K. Templ M. Filzmoser P. Palarea-Albaladejo J. (2015). Bayesian-multiplicative treatment of count zeros in compositional data sets. Stat. Model. 15, 134–158. doi: 10.1177/1471082X14535524

[ref41] Matamoros C. Klopp R. N. Moraes L. E. Harvatine K. J. (2020). Meta-analysis of the relationship between milk *trans*-10 C18:1, milk fatty acids <16 C, and milk fat production. J. Dairy Sci. 103, 10195–10206. doi: 10.3168/jds.2019-18129, PMID: 32921467 PMC7885267

[ref42] Palarea-Albaladejo J. Martín-Fernández J. A. (2015). zCompositions — R package for multivariate imputation of left-censored data under a compositional approach. Chemom. Intell. Lab. Syst. 143, 85–96. doi: 10.1016/j.chemolab.2015.02.019

[ref43] Piperova L. S. Sampugna J. Teter B. B. Kalscheur K. F. Yurawecz M. P. Ku Y.. (2002). Duodenal and milk trans octadecenoic acid and conjugated linoleic acid (CLA) isomers indicate that postabsorptive synthesis is the predominant source of cis-9-containing CLA in lactating dairy cows. J. Nutr. 132, 1235–1241. doi: 10.1093/jn/132.6.1235, PMID: 12042439

[ref44] Pitta D. W. Indugu N. Vecchiarelli B. Hennessy M. Baldin M. Harvatine K. J. (2020). Effect of 2-hydroxy-4-(methylthio) butanoate (HMTBa) supplementation on rumen bacterial populations in dairy cows when exposed to diets with risk for milk fat depression. J. Dairy Sci. 103, 2718–2730. doi: 10.3168/jds.2019-17389, PMID: 31864737

[ref45] Pitta D. W. Indugu N. Vecchiarelli B. Rico D. E. Harvatine K. J. (2018). Alterations in ruminal bacterial populations at induction and recovery from diet-induced milk fat depression in dairy cows. J. Dairy Sci. 101, 295–309. doi: 10.3168/jds.2016-12514, PMID: 29103706

[ref46] Plaizier J. C. Li S. Danscher A. M. Derakshani H. Andersen P. H. Khafipour E. (2017a). Changes in microbiota in rumen digesta and feces due to a grain-based subacute ruminal acidosis (SARA) challenge. Microb. Ecol. 74, 485–495. doi: 10.1007/s00248-017-0940-z, PMID: 28175972

[ref47] Plaizier J. C. Li S. Tun H. M. Khafipour E. (2017b). Nutritional models of experimentally-induced subacute ruminal acidosis (SARA) differ in their impact on rumen and hindgut bacterial communities in dairy cows. Front. Microbiol. 7:2128. doi: 10.3389/fmicb.2016.02128 28179895 PMC5265141

[ref48] Popova M. McGovern E. McCabe M. S. Martin C. Doreau M. Arbre M.. (2017). The structural and functional capacity of ruminal and cecal microbiota in growing cattle was unaffected by dietary supplementation of linseed oil and nitrate. Front. Microbiol. 8:937. doi: 10.3389/fmicb.2017.00937, PMID: 28596764 PMC5442214

[ref49] Quast C. Pruesse E. Yilmaz P. Gerken J. Schweer T. Yarza P.. (2013). The SILVA ribosomal RNA gene database project: improved data processing and web-based tools. Nucleic Acids Res. 41, D590–D596. doi: 10.1093/nar/gks1219 23193283 PMC3531112

[ref50] Quinn T. P. Erb I. Gloor G. Notredame C. Richardson M. F. Crowley T. M. (2019). A field guide for the compositional analysis of any-omics data. Giga Sci. 8, 1–14. doi: 10.1093/gigascience/giz107 PMC6755255 31544212

[ref51] Ren D. Li L. Schwabacher A. W. Young J. W. Beitz D. C. (1996). Mechanism of cholesterol reduction to coprostanol by *Eubacterium coprostanoligenes* a TCC 51222. Steroids 61, 33–40. doi: 10.1016/0039-128X(95)00173-N, PMID: 8789734

[ref52] Rico D. E. Harvatine K. J. (2013). Induction of and recovery from milk fat depression occurs progressively in dairy cows switched between diets that differ in fiber and oil concentration. J. Dairy Sci. 96, 6621–6630. doi: 10.3168/jds.2013-6820, PMID: 23958016

[ref53] Rico D. E. Preston S. H. Risser J. M. Harvatine K. J. (2015). Rapid changes in key ruminal microbial populations during the induction of and recovery from diet-induced milk fat depression in dairy cows. Brit. J. Nutr. 114, 358–367. doi: 10.1017/S0007114515001865, PMID: 26123320

[ref54] Rohart F. Eslami A. Matigian N. Bougeard S. Lê Cao K.-A. (2017a). MINT: a multivariate integrative method to identify reproducible molecular signatures across independent experiments and platforms. BMC Bioinform. 18:128. doi: 10.1186/s12859-017-1553-8, PMID: 28241739 PMC5327533

[ref55] Rohart F. Gautier B. Singh A. Lê Cao K.-A. (2017b). mixOmics: an R package for ‘omics featureselection and multiple data integration. PloS Comput. Biol. 13:1005752. doi: 10.1371/journal.pcbi.1005752 PMC5687754 29099853

[ref56] Tong J. Zhang H. Yang D. Zhang Y. Xiong B. Jiang L. (2018). Illumina sequencing analysis of the ruminal microbiota in high-yield and low-yield lactating dairy cows. PLoS One 13:0198225. doi: 10.1371/journal.pone.0198225 PMC6234037 30423588

[ref57] Toral P. G. Bernard L. Belenguer A. Rouel J. Hervás G. Chilliard Y.. (2016). Comparison of ruminal lipid metabolism in dairy cows and goats fed diets supplemented with starch, plant oil, or fish oil. J. Dairy Sci. 99, 301–316. doi: 10.3168/jds.2015-10292 26601590

[ref58] Toral P. G. Gervais R. Hervás G. Létourneau-Montminy M.-P. Frutos P. (2020). Relationships between *trans*-10 shift indicators and milk fat traits in dairy ewes: insights into milk fat depression. Anim. Feed Sci. Technol. 261:114389. doi: 10.1016/j.anifeedsci.2020.114389

[ref59] Ventto L. Leskinen H. Kairenius P. Stefański T. Bayat A. R. Vilkki J.. (2017). Diet-induced milk fat depression is associated with alterations in ruminal biohydrogenation pathways and formation of novel fatty acid intermediates in lactating cows. Brit. J. Nutr. 117, 364–376. doi: 10.1017/S0007114517000010, PMID: 28236814

[ref60] Wallace R. J. McKain N. Shingfield K. J. Devillard E. (2007). Isomers of conjugated linoleic acids are synthesized via different mechanisms in ruminal digesta and bacteria. J. Lipid Res. 48, 2247–2254. doi: 10.1194/jlr.M700271-JLR200 17644775

[ref61] Weimer P. J. Stevenson D. M. Mertens D. R. (2010). Shifts in bacterial community composition in the rumen of lactating dairy cows under milk fat-depressing conditions. J. Dairy Sci. 93, 265–278. doi: 10.3168/jds.2009-2206, PMID: 20059925

[ref62] Zened A. Combes S. Cauquil L. Mariette J. Klopp C. Bouchez O.. (2013a). Microbial ecology of the rumen evaluated by 454 GS FLX pyrosequencing is affected by starch and oil supplementation of diets. FEMS Microbiol. Ecol. 83, 504–514. doi: 10.1111/1574-6941.12011, PMID: 22974422

[ref63] Zened A. Enjalbert F. Nicot M.-C. Troegeler-Meynadier A. (2013b). A. Starch plus sunflower oil addition to the diet of dry dairy cows results in a *trans*-11 to *trans*-10 shift of biohydrogenation. J. Dairy Sci. 96, 451–459. doi: 10.3168/jds.2012-5690, PMID: 23127910

[ref64] Zened A. Troegeler-Meynadier A. Nicot M.-C. Combes S. Farizon Y. Enjalbert F. (2011). Starch and oil in the donor cow diet and starch in substrate differently affect the in vitro ruminal biohydrogenation of linoleic and linolenic acids. J. Dairy Sci. 94, 5634–5645. doi: 10.3168/jds.2011-4491, PMID: 22032386

