# Supplementary material for: Integrating data from spontaneous and induced trans-10 shift of ruminal biohydrogenation reveals discriminant bacterial community changes at the OTU level
Source: Front Microbiol. 2023 Jan 6;13:1012341. doi: 10.3389/fmicb.2022.1012341 (PMC9853040; doi:10.3389/fmicb.2022.1012341)
Supplement: Supplementary file 1 [file Data_Sheet_1.docx]

**Supplementary tables**

**Supplementary Table 1.** Number of samples, rumen and milk *trans*-10 / *trans*-11 ratio (t10t11R) and milk fat content for the different t10t11R classes .

| class |  | Induced | |  | Field | | |  |
| --- | --- | --- | --- | --- | --- | --- | --- | --- |
|  |  | n = | t10t11R |  | n = | t10t11R | | Milk fat content |
|  |  |  | Rumen |  |  | Rumen | Milk | g/L |
| Low |  | 52 | 0.16 |  | 5 | 0.29 | 0.30 | 37.8 |
| Medium |  | 4 | 0.50 |  | 12 | 0.55 | 0.57 | 34.0 |
| High |  | 7 | 7.97 |  | 5 | 1.94 | 1.86 | 22.6 |

**Supplementary Table 2.** Relative abundance (least squared mean percentages ± SE of GBM transformed values) of bacterial phyla and families with significant differences between trans-10 / trans-11 ratio classes or that significantly correlated with the trans-10 / trans-11 ratio. Statistical analyses were performed on GBM-CLR transformed values.

|  | Low | Medium | High | *P* anova |  | R | *P* correl |
| --- | --- | --- | --- | --- | --- | --- | --- |
| **Phyla** |  |  |  |  |  |  |  |
| Desulfobacterota | 0.08^a^ ± 0.4 | 0.12^ab^ ± 0.34 | 0.76^b^ ± 0.4 | <0.001 |  | 0.34 | 0.013 |
| Proteobacteria | 3.35^a^ ± 2.59 | 8.42^a^ ± 2.39 | 11.66^a^ ± 2.66 | 0.181 |  | 0.28 | 0.036 |
| **Families** |  |  |  |  |  |  |  |
| Eubacteriaceae | 0.01^a^ ± 0.02 | 0.05^b^ ± 0.02 | 0.12^b^ ± 0.02 | <0.001 |  | 0.560 | <0.001 |
| Atopobiaceae | 0.34^a^ ± 0.17 | 0.74^b^ ± 0.22 | 1.83^c^ ± 0.21 | <0.001 |  | 0.558 | <0.001 |
| Veillonellaceae | 0.07^a^ ± 0.22 | 0.36^a^ ± 0.29 | 1.74^b^ ± 0.27 | <0.001 |  | 0.456 | <0.001 |
| Desulfovibrionaceae | 0.08^a^ ± 0.40 | 0.12^ab^ ± 0.34 | 0.76^b^ ± 0.40 | <0.001 |  | 0.338 | <0.001 |
| Bacteroidales RF16 group | 1.16^b^ ± 0.12 | 0.61^a^ ± 0.16 | 0.3^a^ ± 0.15 | <0.001 |  | -0.102 | <0.001 |
| Lachnospiraceae | 12.06^a^ ± 1.59 | 15.95^ab^ ± 2.06 | 20.10^b^ ± 1.94 | <0.001 |  | 0.359 | <0.001 |
| Lactobacillaceae | 0.03^a^ ± 0.03 | 0.03^a^ ± 0.03 | 0.10^a^ ± 0.03 | <0.001 |  | 0.400 | 0.069 |
| Erysipelatoclostridiaceae | 0.02^a^ ± 0.05 | 0.02^ab^ ± 0.05 | 0.14^b^ ± 0.05 | 0.069 |  | 0.212 | 0.004 |
| p-251-o5 | 1.14^b^ ± 0.22 | 0.21^ab^ ± 0.28 | 0.16^a^ ± 0.27 | 0.004 |  | -0.235 | 0.006 |
| Rikenellaceae | 6.50^b^ ± 0.79 | 3.34^ab^ ± 0.94 | 2.22^a^ ± 0.92 | 0.006 |  | -0.224 | 0.010 |
| Paludibacteraceae | 0.06^a^ ± 0.02 | 0.06^a^ ± 0.02 | 0.02^a^ ± 0.02 | 0.010 |  | 0.018 | 0.022 |
| Oscillospiraceae | 4.56^a^ ± 0.45 | 2.59^a^ ± 0.60 | 1.69^a^ ± 0.56 | 0.022 |  | -0.301 | 0.074 |
| Christensenellaceae | 5.66^a^ ± 0.60 | 2.64^a^ ± 0.81 | 1.35^a^ ± 0.76 | 0.074 |  | -0.255 | 0.027 |
| Hungateiclostridiaceae | 0.71^a^ ± 0.08 | 0.62^a^ ± 0.11 | 0.42^a^ ± 0.10 | 0.027 |  | -0.292 | 0.502 |
| F082 | 4.45^a^ ± 0.39 | 2.42^a^ ± 0.50 | 1.72^a^ ± 0.47 | 0.502 |  | -0.191 | 0.035 |
| Spirochaetaceae | 0.37^a^ ± 0.07 | 0.19^a^ ± 0.07 | 0.08^a^ ± 0.08 | 0.035 |  | -0.011 | 0.035 |
| Ruminococcaceae | 7.60^a^ ± 1.26 | 6.56^a^ ± 1.66 | 2.99^a^ ± 1.56 | 0.035 |  | -0.276 | 0.075 |
| Selenomonadaceae | 0.41^a^ ± 0.08 | 0.78^a^ ± 0.10 | 0.13^a^ ± 0.10 | 0.075 |  | 0.092 | 0.042 |

**Supplementary Table 3.** Relative abundance (least squared mean percentages ± SE of GBM transformed values) of genera with significant differences between trans-10 / trans-11 ratio classes or that significantly correlated with the trans-10 / trans-11 ratio. Statistical analyses were performed on GBM-CLR transformed values.

|  | Low | Medium | High | *P* anova |  | R | *P* correl |
| --- | --- | --- | --- | --- | --- | --- | --- |
| **Genera** |  |  |  |  |  |  |  |
| *Syntrophococcus* | 0.24^a^ ± 0.19 | 0.42^a^ ± 0.26 | 1.78^b^ ± 0.24 | <0.001 |  | 0.693 | <0.001 |
| *Olsenella* | 0.19^a^ ± 0.16 | 0.44^b^ ± 0.21 | 1.74^c^ ± 0.20 | <0.001 |  | 0.571 | <0.001 |
| *Pseudoramibacter* | 0.01^a^ ± 0.02 | 0.05^b^ ± 0.02 | 0.12^b^ ± 0.02 | <0.001 |  | 0.560 | <0.001 |
| *[Eubacterium] nodatum* group | 0.07^a^ ± 0.06 | 0.19^b^ ± 0.07 | 0.56^b^ ± 0.07 | <0.001 |  | 0.541 | <0.001 |
| *Shuttleworthia* | 0.24^a^ ± 0.29 | 0.32^a^ ± 0.39 | 1.68^b^ ± 0.36 | <0.001 |  | 0.463 | <0.001 |
| *Dialister* | 0.07^a^ ± 0.22 | 0.36^a^ ± 0.29 | 1.74^b^ ± 0.27 | <0.001 |  | 0.456 | <0.001 |
| *[Eubacterium] cellulosolvens* group | 0.02^a^ ± 0.01 | 0.06^b^ ± 0.01 | 0.05^b^ ± 0.01 | 0.001 |  | 0.440 | <0.001 |
| *Lactobacillus* | 0.03^a^ ± 0.03 | 0.03^a^ ± 0.03 | 0.10^a^ ± 0.03 | 0.071 |  | 0.400 | 0.001 |
| *[Ruminococcus] gauvreauii* group | 0.53^a^ ± 0.26 | 1.43^b^ ± 0.34 | 2.48^b^ ± 0.32 | <0.001 |  | 0.399 | 0.001 |
| *Desulfovibrio* | 0.06^a^ ± 0.40 | 0.10^ab^ ± 0.34 | 0.75^b^ ± 0.40 | <0.001 |  | 0.365 | 0.004 |
| *Roseburia* | 0.09^a^ ± 0.06 | 0.21^ab^ ± 0.07 | 0.42^b^ ± 0.07 | <0.001 |  | 0.361 | 0.004 |
| *[Eubacterium] ruminantium* group | 0.37^a^ ± 0.20 | 0.39^ab^ ± 0.27 | 1.04^b^ ± 0.25 | 0.006 |  | 0.301 | 0.025 |
| *Anaerosporobacter* | 0.08^a^ ± 0.03 | 0.04^a^ ± 0.04 | 0.17^a^ ± 0.03 | 0.135 |  | 0.277 | 0.046 |
| *Succinivibrio* | 0.25^a^ ± 0.52 | 0.75^a^ ± 0.70 | 0.32^a^ ± 0.65 | 0.016 |  | 0.260 | 0.067 |
| *Succinivibrionacea*e UCG-001 | 0.84^a^ ± 2.76 | 5.83^b^ ± 2.45 | 10.34^b^ ± 2.79 | <0.001 |  | 0.250 | 0.071 |
| *Oribacterium* | 0.07^a^ ± 0.19 | 0.29^ab^ ± 0.17 | 0.70^b^ ± 0.19 | <0.001 |  | 0.231 | 0.096 |
| *Erysipelotrichaceae* UCG-002 | 0.02^a^ ± 0.05 | 0.02^ab^ ± 0.05 | 0.14^b^ ± 0.05 | 0.003 |  | 0.212 | 0.122 |
| *Anaerovibrio* | 0.02^a^ ± 0.01 | 0.12^b^ ± 0.02 | 0.01^a^ ± 0.02 | <0.001 |  | 0.148 | 0.357 |
| *Ruminobacter* | 0.20^a^ ± 0.08 | 0.02^a^ ± 0.07 | 0.04^a^ ± 0.08 | 0.045 |  | 0.108 | 0.525 |
| *Selenomonas* | 0.39^a^ ± 0.08 | 0.66^a^ ± 0.11 | 0.09^a^ ± 0.10 | 0.047 |  | 0.045 | 0.833 |
| *Succinivibrionaceae* UCG-002 | 1.63^a^ ± 0.28 | 1.28^a^ ± 0.38 | 0.41^a^ ± 0.35 | 0.035 |  | 0.025 | 0.883 |
| *Papillibacter* | 0.26^b^ ± 0.06 | 0.04^a^ ± 0.08 | 0.03^a^ ± 0.08 | <0.001 |  | -0.332 | 0.011 |
| *Ruminococcus* | 6.40^a^ ± 1.23 | 5.26^a^ ± 1.64 | 2.33^a^ ± 1.53 | 0.042 |  | -0.303 | 0.025 |
| *Saccharofermentans* | 0.68^a^ ± 0.08 | 0.60^a^ ± 0.11 | 0.41^a^ ± 0.10 | 0.482 |  | -0.302 | 0.025 |
| *Christensenellaceae* R-7 group | 5.61^a^ ± 0.60 | 2.62^a^ ± 0.80 | 1.32^a^ ± 0.75 | 0.023 |  | -0.256 | 0.070 |
| *[Eubacterium] ventriosum* group | 0.30^b^ ± 0.07 | 0.20^ab^ ± 0.10 | 0.06^a^ ± 0.09 | 0.009 |  | -0.253 | 0.070 |
| *Lachnospiraceae* XPB1014 group | 0.42^a^ ± 0.08 | 0.18^a^ ± 0.10 | 0.09^a^ ± 0.09 | 0.020 |  | -0.247 | 0.074 |
| *Butyrivibrio* | 0.85^b^ ± 0.11 | 0.38^ab^ ± 0.14 | 0.23^a^ ± 0.13 | 0.023 |  | -0.236 | 0.089 |
| *Rikenellaceae* RC9 gut group | 6.31^b^ ± 0.77 | 3.18^ab^ ± 0.92 | 2.15^a^ ± 0.90 | 0.009 |  | -0.224 | 0.101 |
| *Pseudobutyrivibrio* | 0.45^a^ ± 0.08 | 0.11^a^ ± 0.10 | 0.12^a^ ± 0.10 | 0.014 |  | -0.067 | 0.726 |
| **Species** |  |  |  |  |  |  |  |
| *Olsenella scatoligenes* | 0.02^a^ ± 0.03 | 0.11^b^ ± 0.03 | 0.19^c^ ± 0.03 | <0.001 |  | 0.652 | <0.001 |
| *Eubacterium pyruvativorans* | 0.03^a^ ± 0.05 | 0.11^b^ ± 0.07 | 0.42^c^ ± 0.06 | <0.001 |  | 0.628 | <0.001 |
| *[Eubacterium] cellulosolvens* 6 | 0.02^a^ ± 0.01 | 0.06^b^ ± 0.01 | 0.05^b^ ± 0.01 | <0.001 |  | 0.440 | <0.001 |
| *Prevotella ruminicola* | 0.81^b^ ± 0.09 | 0.38^a^ ± 0.10 | 0.25^a^ ± 0.10 | <0.001 |  | -0.130 | 0.521 |

**Supplementary Table 4.** Excel file. Relative abundance (least squared mean percentages ± SE of GBM transformed values) of OTUs with significant differences between trans-10 / trans-11 ratio classes or that significantly correlated with the trans-10 / trans-11 ratio. Statistical analyses were performed on GBM-CLR transformed values.

**Supplementary figures**

| 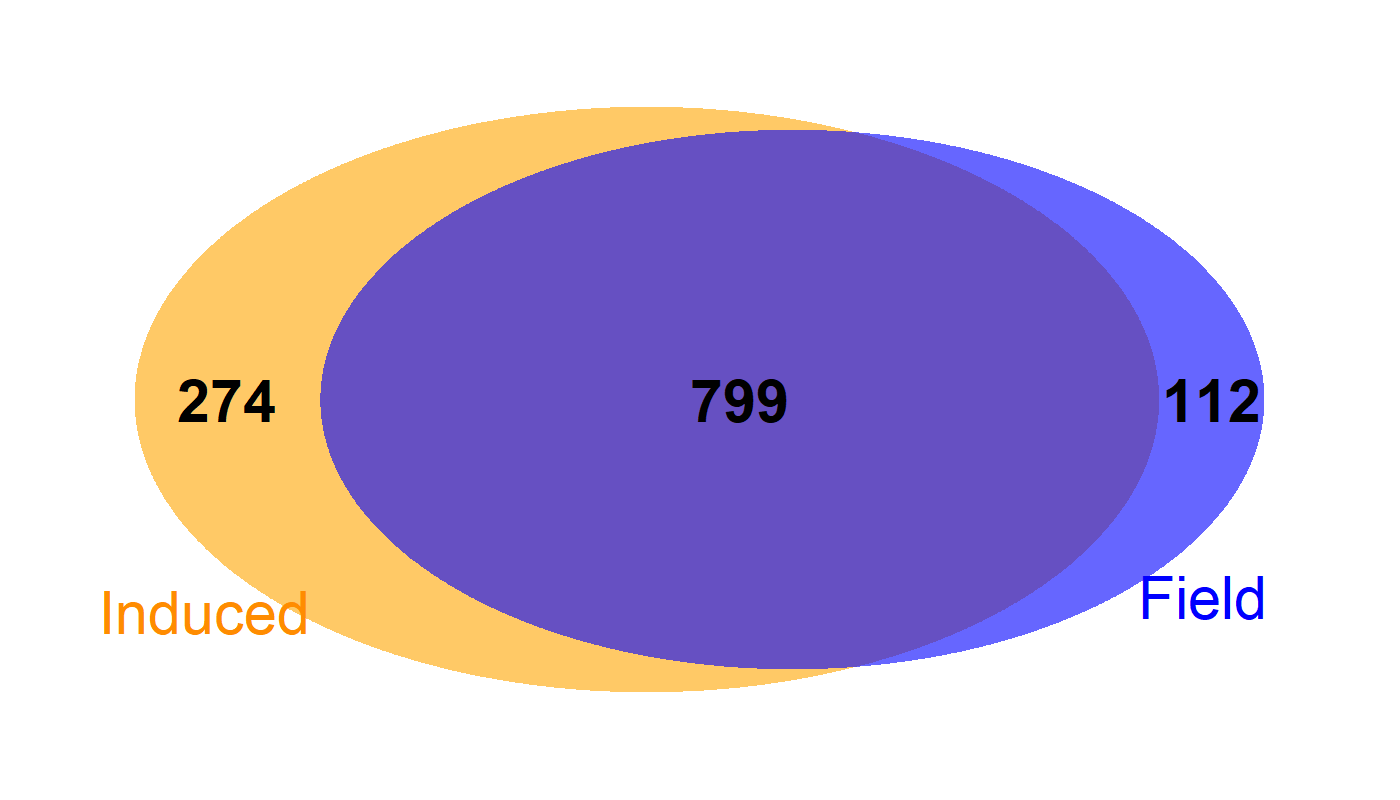 | 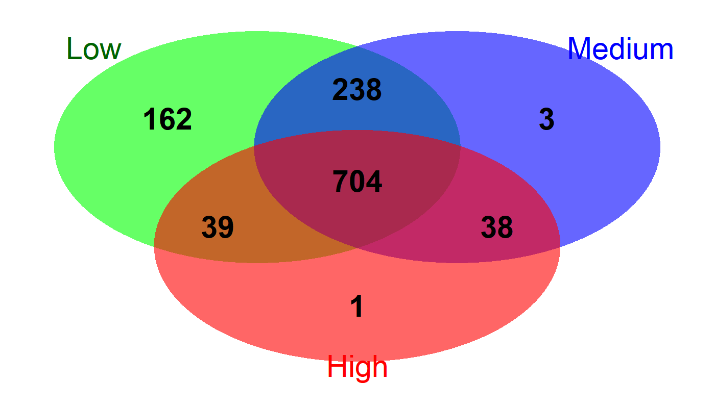 |
| --- | --- |

**Supplementary Figure 1.** Venn diagrams showing the overlaps of OTUs across studies and trans-10 / trans-11 ratio classes.

| 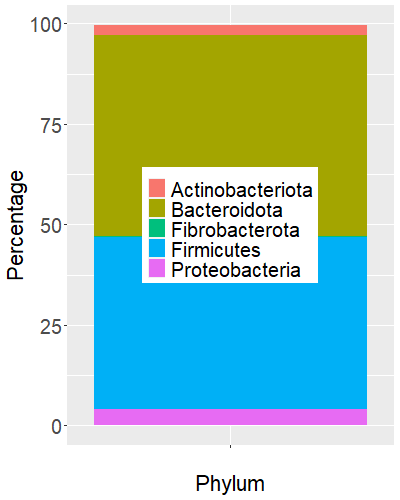 | 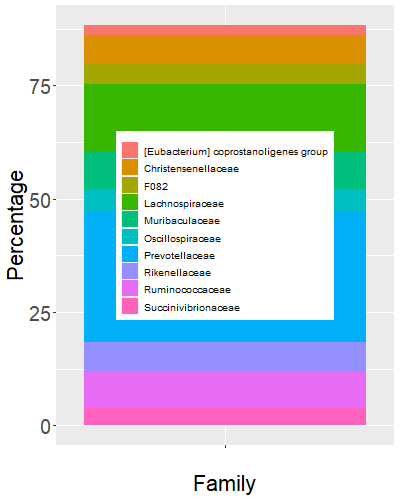 | 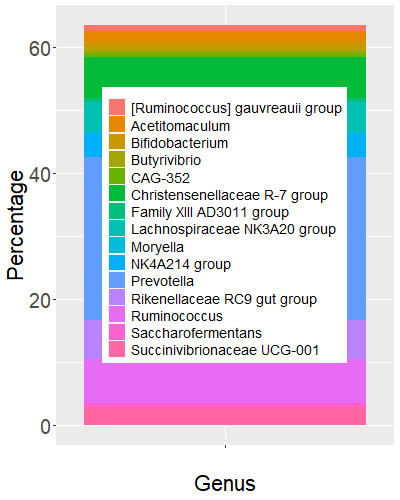 | 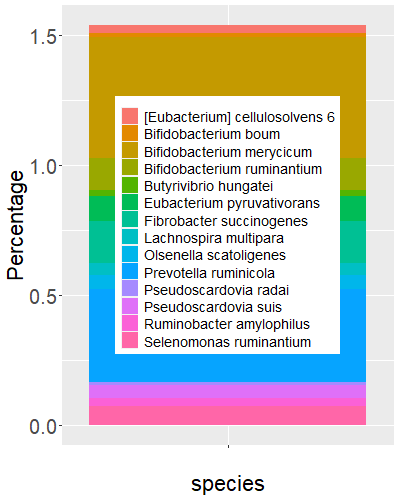 |
| --- | --- | --- | --- |

**Supplementary Figure 2.** Percentage contribution of sequences (%) affiliated at the phylum (5 most abundant), family (10 most abundant), genus (15 most abundant) and species (all identified species) levels.
